# Application of Structural Equation Modeling to determine Emergency Department patient satisfaction drivers

**DOI:** 10.1186/1757-7241-21-S2-A18

**Published:** 2013-09-09

**Authors:** Christian Michel Sørup, Peter Jacobsen

**Affiliations:** 1 DTU Management Engineering, Technical University of Denmark, Denmark

## Background

Diverse theories concerning what emergency department (ED) patients appreciate the most remains a fact despite heavy academic interest during the last decade. Four hypotheses of theoretically grounded causal effects between the latent (unobserved) variables wait time, information delivery, infrastructure and safety are tested by the use of structural equation modeling (SEM).

## Methods

The empirical material is provided by the Unit of Patient-Perceived Quality through a recently published telephone survey. The responses were clustered in categories through an exploratory factor analysis and assessed for construct validity (Cronbach’s alpha). The five hypotheses were analysed further by the use of a two-step structural equation modeling approach as prescribed by Anderson and Gerbing in 1988. First step involves a confirmatory factor analysis to assess validity of a base line model (measurement model) and ensures that the constructs are distinct from each other (discriminant validity). Second step is the alteration of the measurement model into a structural model, which allows for testing of the constructs’ interconnections.

## Results

Two structural models were evaluated for best data fit. The final retained structural model did not dismiss any of the four hypotheses. All path coefficients were statistically significant at a minimum α = 0.05 level, with a single exception.

## Conclusion

Application of SEM on comprehensive empirical data permits clarity of where to target future efforts to improve ED operations. Furthermore, SEM allows for measurement error adjustments and simultaneous estimation of all included parameters. This study manages to extract valuable information in a comprehensive data sample enabled by the application of a mathematically acknowledged modeling technique. Hence, the findings may serve as endorsements for improved ED patient satisfaction rates.

